# Editorial: Next-Generation Sequencing of Human Antibody Repertoires for Exploring B-cell Landscape, Antibody Discovery and Vaccine Development

**DOI:** 10.3389/fimmu.2020.01344

**Published:** 2020-06-30

**Authors:** Ponraj Prabakaran, Jacob Glanville, Gregory C. Ippolito

**Affiliations:** ^1^Biologics Research, Sanofi, Framingham, MA, United States; ^2^Distributed Bio, South San Francisco, CA, United States; ^3^Department of Molecular Biosciences, University of Texas at Austin, Austin, TX, United States

**Keywords:** B-cell receptor repertoire, next generation sequencing, antibodyome, immunoglobulin, B-cell clonotypes, vaccination, immunogenetics, immunoinformatics

The next-generation sequencing (NGS) analysis of human antibody repertoires has enabled a heightened appreciation and comprehensive characterization of the B-cell receptor (BCR) landscape at an unprecedented resolution (1–4). This advance has expanded our insights and lent itself to numerous applications, including the following: NGS coupled with bioinformatics has enhanced phage biopanning of complex antibody libraries and facilitated the antibody discovery process (5); NGS analysis when coupled with large-scale computational structural modeling has revealed sequence and structural correlates between naive and antigen-experienced antibody repertoires (6); and in recent years, NGS-aided study of the antibodyome of HIV-1-infected individuals has increased our understanding of antibody responses and aided the design of antibody lineage-based immunogens that could, in principle, activate naïve precursor B cells to give rise to broadly-reactive neutralizing clones (7, 8). Thus, generally speaking, NGS of human antibody repertoires holds great promise for antibody discovery (9) and vaccine development (10, 11). This editorial introduces 17 high-quality research papers published in the Research Topic which summarize recent developments and applications within the context of NGS analysis of human antibody repertoires, through a combination of Original Research, Methodology, and Review articles.

The topic contains seven Original Research articles. These articles span a wide variety of topics. These studies illustrate means by which to harness the power of NGS for antibody discovery, B-cell immunogenetics, and the evolution of affinity maturation, as well as the investigation of antibody lineages in HIV-1/SIV infections. Hong et al. used cord blood samples from 10 newborn babies and peripheral blood from 33 healthy adults to perform an in-depth analysis of human neonatal and adult IgM heavy chain repertoires. Their comparative study revealed unexpectedly high levels of similarity between the neonatal and adult repertoires although antibody repertoire of healthy adults was more diverse than that of neonates. These results are helpful in understanding the antibody development and diversity in newborn babies and adults. Kirik et al. used NGS to analyze human bone marrow B cells to elucidate how different mutational paths are traversed by antibody lineages stemming from different germline gene origins both in terms of amino acid substitutions, insertions, and deletions. Specifically, they identified germline gene-specific mutational patterns as found in selected and non-selected repertoires. These findings provide a framework for understanding patterns of evolution of antibodies arising from specific, defined germline genes. D'Angelo et al. showed that many different heavy-chain complementarity determining regions 3 (CDR-H3s) could be identified within a target-specific antibody population after *in vitro* selection by using a data set of 32,138 CDR-H3 sequences derived previously from the yeast display sorting and analysis of CDK2-specific antibodies. One of the remarkable observations demonstrated numerous rearranged heavy chains, derived from 19 different germline IGHV genes, were found to contain the same CDR-H3. Their main finding concludes that the same CDR-H3 can be generated by many different rearrangements, but that specific target binding is achieved by only certain unique V-D-J rearrangements and V_L_ pairing. Jeliazkov et al. determined the structural flexibility of the CDR-H3 loops, using previously published algorithms, for thousands of homology models of antibodies derived from the NGS data to find if affinity maturation reduces their conformational flexibility or not. They also used a total of 922 antibody crystal structures from the Protein Data Bank (12) and performed temperature factor analysis and molecular dynamic simulation to assess the flexibility. By using different computational approaches, they came with a conclusion that there is no significant difference between antibody CDR-H3 loop flexibility in repertoires of naïve and mature antibodies. However, they also noted inconsistent results across those methods for some antibodies. They concluded that further experimental methods, for example, hydrogen deuterium exchange mass spectrometry and more accurate modeling or structure determination of antibodies would resolve the inconsistencies. VanDuijn et al. profiled the immune repertoire of rats after immunization with purified antigens using NGS and proteomics. The data obtained from different analysis methods and experimental platforms demonstrate that the immunoglobulin repertoires of immunized animals have overlapping and converging features; however, the quantitative differences between the immune repertoires obtained using proteomic and NGS methods that might relate to differences between the biological niches could not be correlated in this study. With further improvement on the proteomic and NGS immune profiling approaches, their method may enable more interesting applications in biotechnology and clinical diagnostics. Then, He et al. and Han et al. combined the biopanning of scFv phage-displayed antibody libraries and 900 bp long-reads, enabling V_H_/V_L_ paired NGS analysis. He et al. identified broadly neutralizing antibody intermediates from a HIV-1 patient, particularly PGT124 sub-lineage, possessing an invariable CDR-H3 loop and multiple library-derived intermediates, which might serve as a promising template for B-cell lineage vaccine design targeting. Han et al. also showed how they used long-read NGS combined with scFv phage display libraries for identifying SIV gp140-specific antibodies and analyzing their clonotypes and lineages correlating to neutralization activity.

Technical landscape for NGS analysis of human antibodies has changed tremendously and will continue toward the improvement of methods, immunoinformatics and data analysis tools. In this respect, we have four exciting articles devoted to methods/protocols. Hemadou et al. successfully developed, using the PacBio RS II system, and generated long reads (>800 bp) covering full length scFvs following *in vivo* panning in an animal model of atherosclerosis. They tested its performance by tracking and analysis of known, identical and related scFv-phage clone P3. Rosenfeld et al. and Vergani et al. present on a topic of bulk B-cells which provides a way for computationally assessing B-cell clone sizes and a library preparation method for NGS to capture an exhaustive full-length repertoire for nearly every sampled B-cell to be sequenced respectively. Rosenfeld et al. used three different measures of B cell clone size: copy numbers, instances and unique sequences, and then showed how these measures can be used to rank clones, analyze their diversity, and study their distribution within and between individuals. Overall, this method showed how different clone size measures can be used to study the clonal landscape in bulk B cell immune repertoire profiling data. On the other hand, the methodology as adopted by Vergani et al. serves as a useful protocol for Ig-seq where every IGHV-D-J rearrangement in the starting B-cell populations can be detected. Finally, advancements in NGS and error corrections have enabled antibody repertoire sequencing with single mutation precision but still compromising with sequencing accuracy. This opens the possibility for undocumented novel germline alleles. To address on this important issue, Wendel et al. present a method that can be quickly and easily applied to any antibody repertoire data set to mitigate the effects of germline mismatches on SHM patterns.

Next, we provide five excellent reviews in the Research Topic, starting with a review by Chaudhary and Wesemann, which provides a sound introduction to practical steps involved in the process of immune repertoire profiling including sample preparation, platforms available for NGS, sequencing data processing and annotations, and fundamental measurable features of the immune repertoire such as V/D/J gene-segment frequencies, CDR-H3 diversity and physicochemical properties, and immunoglobulin somatic hypermutation (SHM). They also highlight additional analyses using the NGS-derived repertoire data: isotype analysis, which offers insights into the effector biology mediated by heavy chain constant regions, such as complement fixation or binding to Fc receptors; clonal lineage analysis, which is used to trace clonal evolution of HIV-1 broadly neutralizing antibodies; and B-cell network analysis that can link mature antibody sequences to their germline precursor sequences. Extrapolation of these procedures for analyzing paired V_H_:V_L_ repertoires was also discussed. The readers attracted to this review article will likely appreciate the detailed description of statistical tools and their features that can be used for analysis and interpretation of NGS big data sets, along with a comprehensive list of software tools available for sequence error correction, annotation, and evaluation of B cell repertoires. This is followed by a review in which Miho et al. discuss four computational strategies: (i) measuring immune repertoire diversity, (ii) clustering and network approaches to resolve the sequence similarity architecture, (iii) phylogenetic methods to retrace antigen-driven evolution, and (iv) machine learning methods to dissect naïve and antigen-driven repertoire convergence. Furthermore, they summarize outstanding questions in computational immunology and propose new directions for systems immunology by possibly linking NGS-based potential metrics with computational discovery of immunotherapeutics, vaccines, and immunodiagnostics. These two reviews are followed by a mini-review article by Rouet et al., which specifically addresses the strategies for NGS of phage- and other antibody-display libraries, and list NGS platforms and analysis tools. This review also touches briefly on bioinformatic tools and applications to design validation with analyses of naïve antibody libraries, affinity maturation and epitope mapping with specific examples from literature. After these three reviews, our Research Topic addresses a challenging question of how B-cell receptor repertoire sequencing can potentially be enriched when coupled with structural antibody data, as described in the review by Kovaltsuk et al.. This review covers the basic principles about structural architecture of IgG, repertoire sequencing technologies and antibody structural properties. Further, they highlight on computational approaches and tools that leverage antibody structure information and provide a generalized workflow of antibody modeling. Overall, the authors illustrate how these two data types—NGS DNA sequences (i.e., BCR-seq) and atomic structures, that can enrich one another and yield potential for advancing our knowledge of the immune system and improving antibody engineering and developability. Along this line of work, Mishra and Mariuzza review the structural basis of antibody affinity maturation from NGS data. Interestingly, they looked at the studies of antibody affinity maturation prior to and after NGS. They further emphasized how important the NGS is for the reconstruction of antibody clonal lineages in immune responses to viral pathogens, such as HIV-1. They discussed in detail about various mechanisms of paratope preorganization, rigidification, reorientation, and indels as described for many antibodies. Overall, this review provides a more holistic perspective to structural basis of antibody affinity maturation from the point of next-generation sequencing.

To finish this topic, we aptly include a perspective article on reproducibility and reuse of adaptive immune receptor repertoire data. We are delighted to have included an excellent contribution from the Adaptive Immune Receptor Repertoire (AIRR) community (Breden et al.), which provides an overview of the founding principles and presents the progress it has made to develop and promote standards and recommendations for best practices and data-sharing protocols. In conclusion, NGS combined with innovative single-B-cell technologies has the potential to yield millions of native human antibody sequences and some of them that could match with therapeutic antibodies (13, 14). This suggests a possible implication for data mining in the NGS repositories for discovering therapeutic antibody candidates in future. Also, large-scale NGS analysis of individual antibodyome will lead to improved insights into overall diversity of the human antibody repertoire and B cell immunogenetics (15–17).

## Author Contributions

PP wrote the manuscript. All authors contributed to this work and approved the final version of the manuscript.

## Conflict of Interest

PP is an employee of Sanofi Genzyme. JG is an employee and CEO of Distributed Bio. The remaining author declares that the research was conducted in the absence of any commercial or financial relationships that could be construed as a potential conflict of interest. The handling editor declared a past co-authorship with one of the authors GI.
